# Development of Sign Language Motion Recognition System for Hearing-Impaired People Using Electromyography Signal

**DOI:** 10.3390/s20205807

**Published:** 2020-10-14

**Authors:** Shigeyuki Tateno, Hongbin Liu, Junhong Ou

**Affiliations:** Graduate School of Information, Production and Systems, Waseda University, Kitakyushu 808-0135, Japan; liuhongbin@toki.waseda.jp (H.L.); ohjunhong@ruri.waseda.jp (J.O.)

**Keywords:** motion recognition, electromyography, long short-term memory neural network, bilinear model, sign language

## Abstract

Sign languages are developed around the world for hearing-impaired people to communicate with others who understand them. Different grammar and alphabets limit the usage of sign languages between different sign language users. Furthermore, training is required for hearing-intact people to communicate with them. Therefore, in this paper, a real-time motion recognition system based on an electromyography signal is proposed for recognizing actual American Sign Language (ASL) hand motions for helping hearing-impaired people communicate with others and training normal people to understand the sign languages. A bilinear model is applied to deal with the on electromyography (EMG) data for decreasing the individual difference among different people. A long short-term memory neural network is used in this paper as the classifier. Twenty sign language motions in the ASL library are selected for recognition in order to increase the practicability of the system. The results indicate that this system can recognize these twenty motions with high accuracy among twenty participants. Therefore, this system has the potential to be widely applied to help hearing-impaired people for daily communication and normal people to understand the sign languages.

## 1. Introduction

According to the World Health Organization, 466 million people are suffering from hearing loss around the world in 2020 [1]. Sign language is an essential tool for them to communicate with others. Recently, studies of deafness have adopted more complex sociocultural perspectives, raising issues of community identity, formation and maintenance, and language ideology [2]. As a meaning to construct individual communication, sign languages do not share the same standard worldwide. Instead, cultural difference along with other factors create a huge difference among the sign languages [3,4,5,6]. The physical component of sign languages usually consists of movement of forearm and hand motion. The formation of sentences is also different in terms of grammar, vocabulary, and alphabets among various sign languages [7]. Obstacles stand between hearing-intact people and hearing-impaired people when communicating; therefore, special training is required to understand the sign languages.

Different approaches are applied to develop a sign language recognition system in which camera sensors and sensor-integrated gloves are commonly used. In 2014, C. H. Chuan et al. used the leap motion sensor to capture the hand movements of the user to recognize different hand gestures of the American Sign Language (ASL) [8,9]. H. E. Hayek et al. proposed a sign translator system by using a hand glove in 2015 [10]. However, in some situations, limitations of these sensors exist. The camera sensor requires a lighting environment and has a limited detection range [11,12], and the glove is expensive and uneasy to be worn [13,14].

In order to solve the problems, researchers choose a kind of sensor based on electromyography (EMG) signal. The EMG signal collects bioelectrical signals of muscles during muscle extension or contraction from the forearm, which can avoid limitations of the camera sensors and the glove sensors. The EMG signal from the forearm can be utilized to control an artificial arm [15,16]. Moreover, functional states of muscle movements can be reflected by the EMG signal [17,18,19,20,21]. Savur and Sahin used a surface EMG signal to recognize the ASL letters alphabet to allow users to spell words and sentences with an accuracy of 60% [22]. Lionel and other researchers used convolutional neural networks (CNNs) on the EMG data of the forearm to recognize 20 Italian gestures [23]. Seongjoo and others used sensor fusion technology and group-dependent neural network models to recognize Korean sign language [24]. After the collection of EMG data, measures are applied to process raw EMG data. Features of the EMG data can be obtained by applying a model on each channel or modeling all channels as a whole [25]. In both the time domain and frequency domain, features are extracted to analyze the EMG data [26,27]. Recently, some studies are focusing on transforming time-series EMG data into images for utilizing image recognition techniques to avoid information loss during the feature extracting process [28,29]. Various classifiers are applied to identify different movements of muscles [30,31,32].

In essence, the sign language recognition system is to distinguish the time continuous gestures of forearms. Jaramillo-Yánez and others made a systematic review on this subject [33]. Various devices of different sampling rate ranging from 100 Hz to 1200 Hz were used to collect raw data [34,35,36,37]. Approximately 95% of the signal power was below 400–500 Hz, which required the sampling rate to reach 1000 Hz in order to gather all the information according to the Nyquist sampling theory [38,39,40]. However, studies employing a low sampling rate device still obtained a decent accuracy with different approaches applied [41,42,43]. Some of other studies only carried the experiment on single participant and developed systems which had high accuracy [44,45,46]. However, as an electrophysiological signal, the EMG signal has individual differences, which will greatly reduce the accuracy of the system based on a single participant [47]. The same movements of the same muscles in different people can generate different EMG signals. Several methods were proposed to tackle this problem [48,49,50,51]. Some researchers developed a bilinear model to overcome individual differences. In 2000, J. B. Tenenbaum et al. proposed the definition and the algorithm of a bilinear model to deal with the problem of face recognition [52]. In 2013, Matsubara et al. applied the bilinear model for the first time in the recognition of five types of hand gestures (four types of motion gestures and one type of static relaxation) to control a robot hand [53]. Later, in 2014, Wang Tao et al. used a bilinear model to perform a single-finger compression experiment under different contractions [54].

Moreover, the review [33] showed that almost all the studies considered improving recognition accuracy and a few studies considered implementing actual real-time application on portable devices or embedded systems. In our research, a sign language motion recognition system based on electromyography (EMG) signal is proposed to realize a real-time application. Conventional EMG data processing methods are utilized to extract ten features from raw EMG data, and then the constructed features are input into a bilinear model. Conventional feature processing includes the feature dimensionality reduction and normalization. By extracting the features that have more contributions like the principal component analysis, as well as normalization of feature amplitude or time scale, individual differences can be eliminated within a certain range [55]. In addition, using a small number of training samples of test users to participate in the learning and training of the classifier can improve the recognition results of non-specific persons to a certain extent, like the transfer learning method [56]. By inputting the features into the bilinear model, the relationship between users and motions is considered. Thus, an EMG control interactive system with the ability to recognize non-specific human motions with high accuracy is constructed. By using this system, the sign language motions which are commonly used in the daily life of hearing-impaired people can be recognized in real time and the meaning of these motions can be output to make normal people understand.

## 2. Mechanism and Algorithm

In this proposed system, the EMG signal of muscle is obtained to classify into different motion categories. A long short-term memory (LSTM) neural network is utilized as a classifier since the LSTM has good performance in time-series data classification. The memory units in the LSTM can be of great help in maintaining the useful information and discarding the interferences from the previous input data to affect the current state positively.

**Figure 1:**
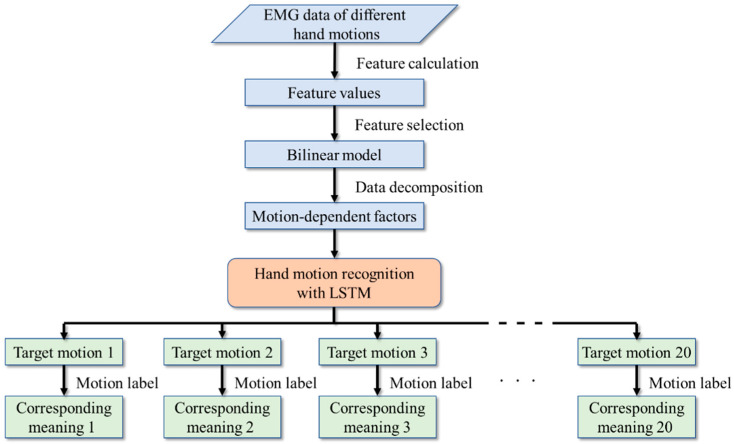
The flow chart of the system.

Firstly, the armband is worn on the user’s forearm. During the process of making hand motions, the surface EMG signal is recorded to analyze the information of the muscles, such as muscle contraction, extension, and relaxed. Secondly, several widely used features are calculated to obtain the characteristics of the EMG data in both the time domain and the frequency domain. The feature selection needs to be applied to reduce the computation cost of the system. Therefore, the permutation feature importance algorithm [57] is conducted, and then several useful features will be selected. Thirdly, the parameters of a bilinear model will be adjusted, then the selected feature values are decomposed by the bilinear model for extracting the motion-dependent factors to decrease the individual difference of the EMG data since it differs obviously among different people. Fourthly, the obtained motion-dependent factors are input into the LSTM for recognition. Finally, the motion label of the EMG data is used to output the corresponding meaning of the hand motion. The flow chart of the whole system is shown in Figure 1.

### 2.1. EMG Data Collection

**Figure 2:**
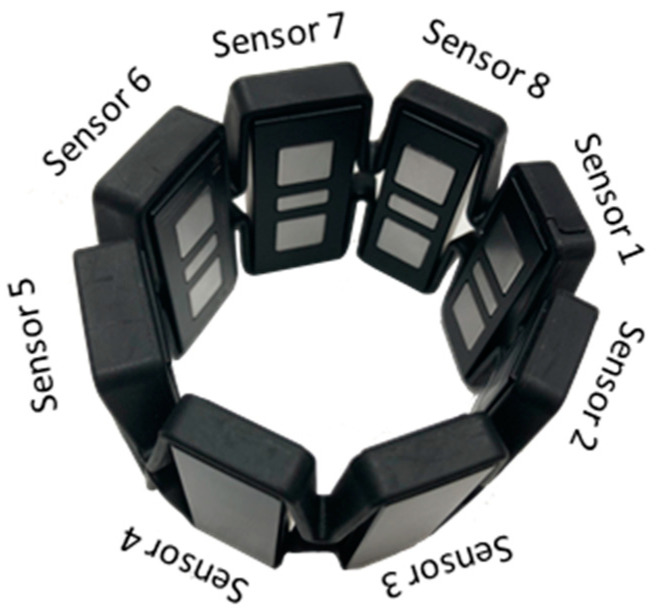
The Myo armband.

The EMG sensor applied in this system is a Myo armband which is manufactured by Thalmic Labs. Compared with other EMG sensors such as electrodes made by Delsys and Otto Block company, the Myo armband transmits EMG data through Bluetooth, which can ensure the quality of EMG signals by reducing noise caused by cables and make it easy to wear. The Myo armband shown in Figure 2 has eight EMG sensors whose sampling rate is 200 Hz.

**Figure 3:**
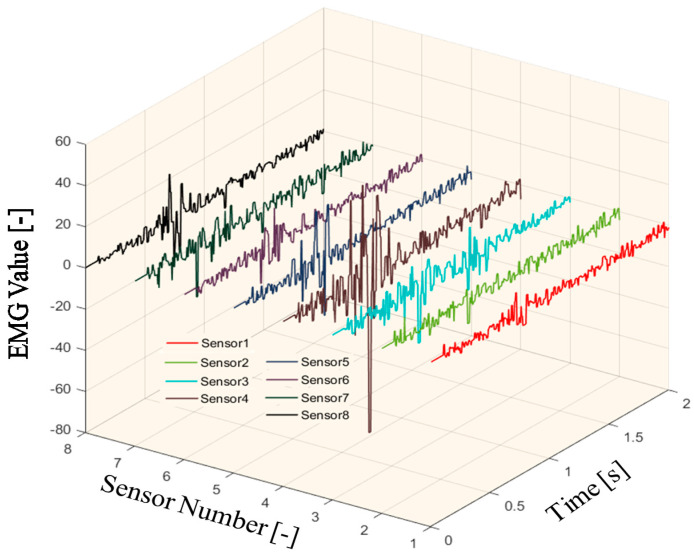
The electromyography (EMG) data in eight channels.

An example of the EMG data in eight channels is shown in Figure 3.

### 2.2. EMG Data Processing

Since the EMG data provided by the armband is the time-series data that describes how the muscle state varies during the process of performing hand motions, conventional data processing methods are selecting suitable features in both time and frequency domains to calculate the feature values as the input of classifiers. 

The feature extraction is an important method to obtain useful characteristics of the EMG data and remove redundant or interfering information. Sometimes, the feature dimensionality reduction and the permutation feature importance are needed to select more important information from the feature values. Here, some commonly used features are listed as shown in Equations (1)–(10).

The first part is about the time-domain features which are calculated based on the raw time series EMG data. As the most basic and commonly used feature in statistical analysis, the mean absolute value (MAV) is calculated as:

(1) $$MAV_{j}=\frac{1}{N}\sum_{i=1}^{N}|{\left(EMG_{i}\right)}_{j}|,\;j=1,2,\ldots ,C,$$

where *j* is the channel number of the EMG data, ${\left(EMG_{i}\right)}_{j}$ is the single value of the EMG data in channel *j*, *N* is the amount of the EMG data in channel *j*.

The second one is the standard deviation (STD), which describes the value variation of the EMG data:

(2) $$STD_{j}=\sqrt{\frac{1}{N}\sum_{i=1}^{N}\left({\left(EMG_{i}\right)}_{j}\right)-\mu {)}^{2}},\;j=1,2,\ldots ,C,$$

where μ is the average value of the EMG data in channel *j*.

The third one is the root mean square (RMS). In the EMG analysis area, it is modeled as an amplitude modulated Gaussian random process, which relates to constant force and non-fatiguing contraction [58]. The RMS is calculated as follows:

(3) $$RMS_{j}=\sqrt{\frac{1}{N}\sum_{i=1}^{N}{\left[{\left(EMG_{i}\right)}_{j}\right]}^{2}},\;j=1,2,\ldots ,C.$$

The fourth one is the log detector (LOG), which provides the estimations of muscle contraction force [59], as shown in Equation (4).

(4) $$LOG_{j}=e^{\frac{1}{N}\overset{N}{\underset{i=1}{\sum }}log\left|{\left(EMG_{i}\right)}_{j}\right|},\;j=1,2,\ldots ,C.$$

The final one is the average amplitude change (AAC), which is a measurement of the complexity of the EMG data and represents the average of the data difference over the time segment [59]. It can be calculated as:

(5) $$AAC_{j}=\;\frac{1}{N-1}\sum_{i=1}^{N-1}|{\left(EMG_{i+1}\right)}_{j}-{\left(EMG_{i}\right)}_{j}|,\;j=1,2,\ldots ,C.$$

**Figure 4:**
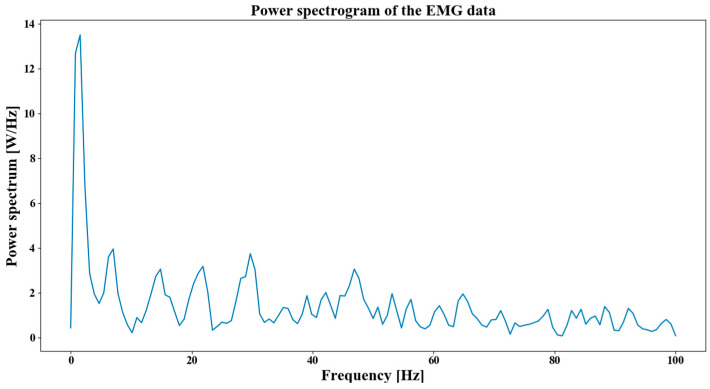
The power spectrogram of EMG data.

The second part is the frequency-domain features that represent the generated power of the working muscle during muscle movements and can be used to detect muscle fatigue. In order to obtain the frequency-domain features, the power spectrogram of the EMG data is firstly calculated which is based on the Welch’s method. The data used in this research is obtained from a Myo band with a sampling rate of 200 Hz. The result is shown in Figure 4.

Then, the first frequency-domain feature is the mean frequency (MNF) which is the sum of the product of the EMG power spectrum and the frequency divided by the sum of spectrum intensity, as shown in Equation (6):

(6) $$MNF_{j}=\sum_{i=1}^{N}f_{i}P_{i}/\sum_{i=1}^{N}P_{i},\;j=1,2,\ldots ,C.$$

where $f_{i}$ is the frequency of the spectrum at the *i*-th frequency bin after the Fourier transform, $P_{i}$ is the *i*-th spectrum value and *N* is the length of the frequency bin.

The second commonly used frequency-domain feature is the median frequency (MDF), which divides the spectrums into two regions with equal amplitude:

(7) $$\sum_{i=1}^{MDF}P_{ij}=\sum_{MDF}^{N}P_{ij}=\frac{1}{2}\sum_{i=1}^{N}P_{i},j=1,2\ldots ,C.$$

Similar to the MNF, the mean power (MNP) is calculated as follows:

(8) $$MNP_{j}=\sum_{i=1}^{N}f_{i}P_{i}/\sum_{i=1}^{N}f_{i},\;j=1,2,\ldots ,C.$$

The next one is the power spectrum ratio (PSR), which measures the ratio between the maximum value and the whole energy of the EMG power spectrum:

(9) $$PSR_{j}=\;\frac{P_{0}}{P}=\frac{\sum_{f_{0}-\varepsilon }^{f_{0}+\varepsilon }P_{i}}{\sum_{f_{1}}^{f_{2}}P_{i}},\;j=1,2,\;\ldots ,\;C.$$

The last frequency-domain feature is the peak frequency (PKF), which is the frequency value where the maximum power value appears:

(10) $$PKF_{j}=f_{j}\;\left(max\left(P_{i}\right)\right),\;j=1,2,\;\ldots ,C.$$

After the feature calculation, the calculated feature values are firstly selected by a permutation feature importance process, and later input into a bilinear model to extract the motion-dependent factors for classification.

### 2.3. Bilinear Model Algorithm

**Figure 5:**
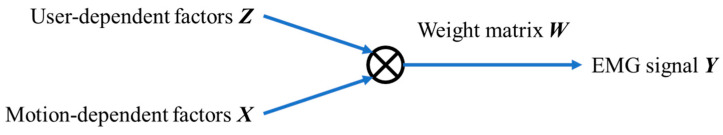
The composition of the EMG signal in the bilinear model.

According to the definition of the bilinear model, the EMG signal ***Y*** can be decomposed into the user-related factors ***Z*** and the motion-related factors ***X*** with a weight matrix ***W*** to describe the factor interactions, as shown in Figure 5 [52,53,54].

For a single EMG signal value *y*, it can be represented in the following form:

(11) $$y=z{\;}^{T}W_{c}x,$$

where $z\in R^{I}$ represents the user-related factor and $x\in R^{J}\;$ represents the motion-related factor. $W_{c}\in R^{I*J}$ is the parameter matrix of the bilinear model which describes the factor interactions between z and x.

Suppose that the EMG signal is ***Y*** $\in R^{C}$ where c∈1~C is the channel serial number of the EMG signal, the subject serial number is u∈1~U the motion serial number is m∈1~M and the data serial number for one motion is n∈1~N Therefore, the problem of fitting the bilinear model can be described as searching for suitable variables {$z^{uT},W_{c},x_{n}^{m}$} for all *u*, *c*, *m*, and *n*, to minimize the difference between the constructed EMG signal which is calculated by Equation (11) and the original EMG signal ***Y***. Therefore, the objective function of fitting the bilinear model is as follows:

(12) $$E=\sum_{u=1}^{U}\sum_{n=1}^{N}\sum_{m=1}^{M}\sum_{c=1}^{C}||y_{cn}^{um}-z^{uT}W_{c}x_{n}^{m}|{|}^{2}\;.$$

Conventionally, the definition of the EMG signal is a multiple-dimensional matrix, in which each dimension describes different information such as users, channels, motions, and so on, respectively. However, in the bilinear model, the matrices are always two-dimensional in order to utilize some standard matrix processing algorithms. Therefore, the multiple-dimensional matrices are expanded into the stacked two-dimensional matrices where the data is arranged in a specific order. By using this concept, the obtained dataset of EMG signal ***Y*** can be presented in the following form:

(13) $$Y=[\begin{array}{llllll} y_{{}_{11}}^{11} & \ldots & y_{{}_{1N}}^{11} & {\;y}_{{}_{11}}^{12} & \ldots & y_{{}_{1N}}^{1M}\\ \vdots & & \vdots & \vdots & & \vdots \\ y_{{}_{C1}}^{11} & \ldots & y_{{}_{CN}}^{11} & {\;y}_{{}_{C1}}^{12} & \ldots & y_{CN}^{1M}\\ y_{{}_{11}}^{21} & \ldots & y_{{}_{1N}}^{21} & {\;y}_{{}_{11}}^{22} & \ldots & y_{{}_{1N}}^{2M}\\ \vdots & & \vdots & \vdots & & \vdots \\ y_{C1}^{U1} & \ldots & y_{CN}^{U1} & {\;y}_{C1}^{U2} & \ldots & y_{{}_{CN}}^{UM} \end{array}]\in R^{CU*MN}$$

where *U* is the number of users, *C* is the number of channels, *M* is the number of motions, and *N* is the amount of data in one motion. Similarly, the definitions of the user-related matrix ***Z***, the motion-related matrix ***X***, and the weight matrix ***W*** are shown from Equations (14) to (16):

(14) $$Z=\left[\begin{array}{ccc} z^{1}, & z^{2}, & \begin{array}{cc} \cdots & z^{U} \end{array} \end{array}\right]\in R^{I*U}\;,$$

(15) $$X=\left[\begin{array}{ccc} x_{1}^{1}, & x_{2}^{1}, & \begin{array}{cc} \cdots & \begin{array}{ccc} x_{N}^{1}, & \cdots & x_{N}^{M} \end{array} \end{array} \end{array}\right]\in R^{J*MN}\;,$$

(16) $$W=\left[\begin{array}{ccc} W_{1}, & W_{2}, & \begin{array}{cc} \cdots & W_{C} \end{array} \end{array}\right]\in R^{IC*J}\;.$$

**Figure 6:**
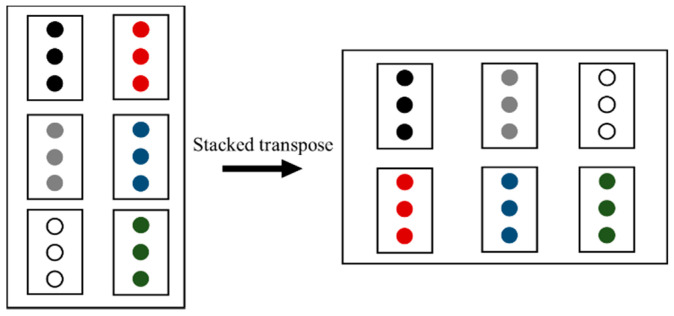
The Schematic diagram of the stacked transpose.

Basically, the calculation methods of the stacked matrices are the same as normal matrices. However, there are still some differences. When the channel number of the EMG signal is larger than one, the data of the same user and motion but different channels should be considered as a whole part for the calculation, especially for the matrix transpose. Therefore, the stacked transpose (ST) is defined as follows: for a MC×N stacked matrix, its ST can be defined as a NC×M matrix, as shown in Figure 6.

With all these definitions, two equivalent equations of the variables introduced above can be obtained as shown from Equations (17) and (18):

(17) $$Y=[W{\;}^{\mathrm{ST}}Z]{\;}^{\mathrm{ST}}X,$$

(18) $$Y^{ST}=\left[WX\right]{\;}^{\mathrm{ST}}Z.$$

For determining the optimal matrices ***Z***, ***X***, and ***W***, the iterative procedure is as follows. Firstly, the singular value decomposition (SVD) of the EMG signal ***Y*** is calculated as $Y\overset{SVD}{\to }U\sum^{\;}V^{T}$ where U and $V^{T}$ are unitary matrices and ∑ is a diagonal matrix whose diagonal elements are the singular values of the ***Y***. Then, ***X*** is initialized as the first *J* rows of the $V^{T}$. Next, from the initialized ***X*** and the EMG data ***Y***, ***Z*** is updated as the first *I* rows of the $V^{T}$, where $[YX^{T}]{\;}^{\mathrm{ST}}\overset{SVD}{\to }U\sum^{\;}V^{T}$ Finally, using the derived ***Z*** and the EMG data ***Y***, updating the ***X*** as the first *J* rows of the $V^{T}$, where ${\left[Y^{\mathrm{ST}}Z^{T}\right]}^{\mathrm{VT}}\overset{SVD}{\to }U\sum^{\;}V^{T}$ Except for the initialization of the ***X***, the updating procedure of the ***Z*** and the ***X*** is a one-time iteration of the bilinear model algorithm. This algorithm converges within 10 iterations. Once the algorithm converges, the obtained matrix ***X*** of which each component $x_{n}^{m}$ corresponds to the motion label *m* is used for training a classifier. 

For testing the performance of the bilinear model, a new subject needs to perform at least one motion  m to extract his/her user-related matrix $z_{new}$ with Equation (19):

(19) $$\;z_{new}\;=\left\{\left[{\left[WX_{m}\right]}^{\mathrm{ST}}\;\right]\right\}{\;}^{+}\;*\;Y_{new\_m}{}^{\mathrm{ST}}\;,)$$

where $\;{\left\{\cdot \right\}}^{+}$ means the pseudo-inverse matrix of matrix {·},$\;Y_{new\_m}$ is EMG data of the new subject when performing the motion *m*, and ***W*** and $X_{m}$ are previous weight matrix and the corresponding motion matrix from the obtained matrix ***X***, respectively. With the new user-related matrix $z_{new}$ the new weight matrix $W_{new\_m}$ is derived by Equation (20):

(20) $$W_{new\_m}=\;{\left\{{\left[\;Y_{new\_m}{}^{\mathrm{ST}}{\left\{z_{new}\right\}}^{+}\right]}^{\mathrm{ST}}\;\right\}}^{+}\;\times {\left\{X_{m}\right\}}^{+}\;.$$

Finally, with the derived $z_{new}$ and $W_{new\_m}$ a new motion-related matrix $X_{new\_m}$ for motion *m* is extracted by Equation (21):

(21) $$X_{new\_m}=\;\left\{{\left[W_{new\_m}{}^{\mathrm{ST}}z_{new}\right]}^{\mathrm{ST}}\;\right\}{\;}^{+}\;\times \;Y_{new\_m}\;.$$

The new motion-related matrix $X_{new\_m}$ of which each component $x_{new}{}_{n}^{m}$ corresponds to the motion label *m* is obtained for testing the classifier.

### 2.4. Hand Motion Classify

In this paper, an LSTM is chosen as the hand motion classifier. In order to introduce the LSTM, firstly, the concept of a recurrent neural network (RNN) needs to be clarified. An RNN is a kind of deep neural network whose current node weight is not only decided by the current input but also affected by the previous input [60]. Therefore, the RNN is widely applied to deal with the data which can be considered to be related in time slices; that is, the state generated at the current time point is affected by the previous time point, and it will affect the output state at the subsequent time point. 

**Figure 7:**
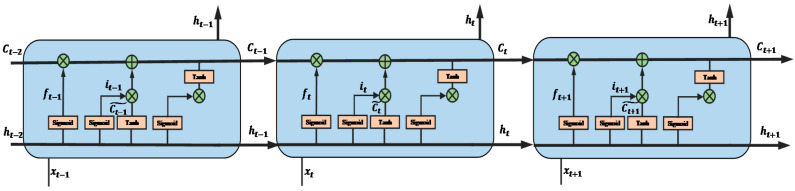
The structure of the long short-term memory (LSTM).

However, for a long time-series data, since the structure of the RNN is essentially a recursive nested structure, which will cause the problem of “gradient explosion” or “gradient vanish” so that all the previous information is lost [60]. Therefore, the LSTM is introduced to add mainly three gates to control the information circulation: how much previous information dropping, how much current information inputting, and how much current information outputting. With these gates controlling the information circulation, the performance of the LSTM is much better than the simple RNN in a long time-series data classification task. Therefore, in this paper, the LSTM is selected as the classifier. The structure of the LSTM is shown in Figure 7.

## 3. Experiment

The experiment consisted of two steps. The first step was to carry the experiment on a single participant. The second step was to experiment with multiple participants.

**Figure 8:**
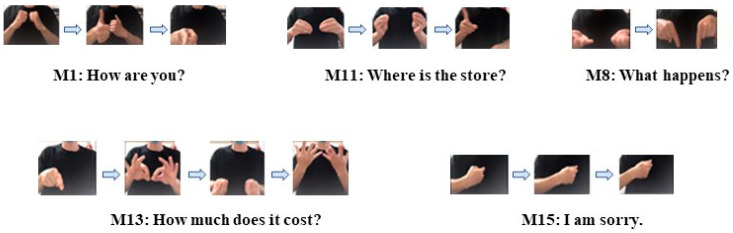
Sign language motions.

For the first step, the first thing to confirm was that the EMG signal could be used to recognize different hand motions for one participant. According to the ASL library, 20 hand motions of different meanings that are used for actual communication by hearing-impaired people were selected for recognition. The 20 hand motions are shown in Table 1. Some examples are shown in Figure 8.

**Figure 9:**
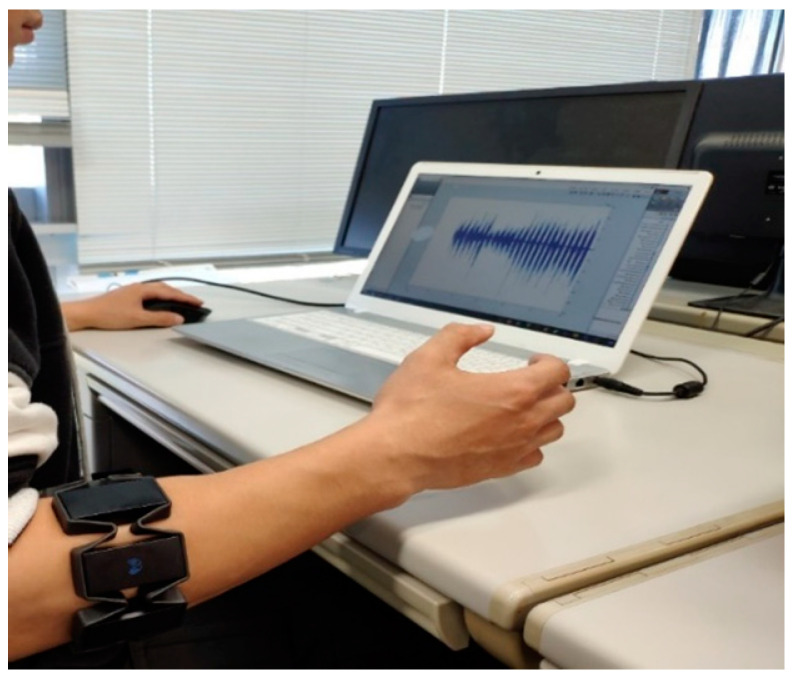
The environment settings of EMG data obtaining.

The participant was asked to wear the armband on his right hand since the selected 20 motions include the right-hand motions and the participant was right hand dominated. The experiment environment setting is shown in Figure 9.

At the beginning of the experiment, the participant was asked to watch the videos of the sign language motions recorded in advance and practiced the motions in order to finish the motion within three seconds. What is more, before the experiment day, the participant was asked not to conduct heavy activities that require the right hand in dominate to avoid the muscle fatigue of his forearm.

For the second step, in order to apply the system to a wide range of users, the experiments on multiple participants were conducted. Twenty healthy male participants whose ages are between 23 and 25 and whose dominant hand is the right hand are recruited. For each participant, each hand motion is performed within three seconds, and each experiment was conducted under the same condition and environment as the participant one.

## 4. Results

### 4.1. Single-Person Experiment

**Figure 10:**
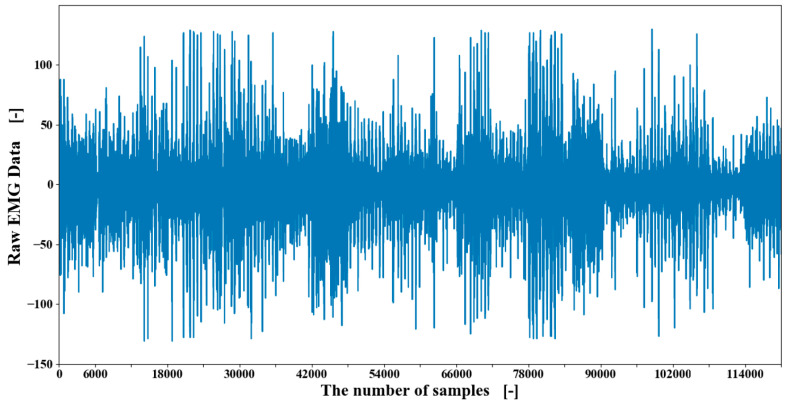
The obtained raw EMG data of 20 motions of participant one (10 times repeating for each motion).

In this experiment, the participant was required to perform each motion within three seconds which was corresponding to 600 pieces of EMG data. For each motion, the repeating cycle was set as 10, and moreover this cycle was repeated five times, changing the sensor position to obtain enough EMG data as much as possible in order to perform the permutation importance method. The obtained EMG data of one participant is shown in Figure 10.

**Figure 11:**
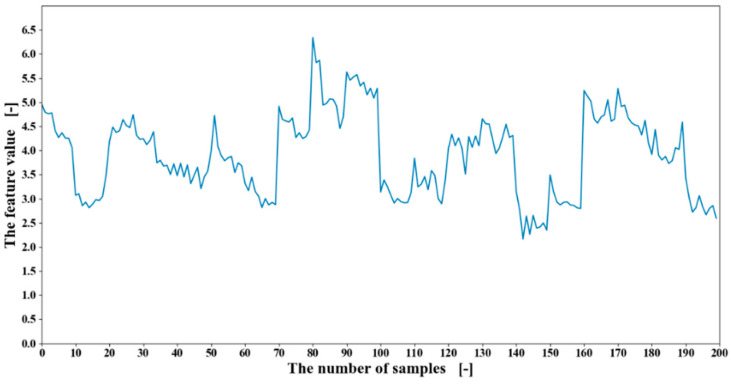
The root mean square (RMS) feature value of one channel from obtained EMG data of participant one.

The time-domain features and the frequency-domain features introduced in Section 2.2 were calculated with the window size of 600. Therefore, for each motion, 20 feature stacks were obtained. For example, the calculated RMS feature values from one channel of the EMG data of participant one are shown in Figure 11. Each motion was performed 50 times, and there were 20 motions in total.

First, all the 10 features were used for training and testing of the LSTM. In the LSTM, after using different learning rates ranging from 0.00001 to 0.001, the learning rate is set to 0.0001 for higher accuracy possible. The iteration time is set to 500 which can make the validation loss converge on the training dataset. The training dataset and the testing dataset are with a ratio of 0.8 and 0.2, respectively. Each motion is performed totally 50 times. The results of the LSTM are shown in Table 2. 

The result of permutation feature importance is shown in Table 3. From Table 3, the priority of the features based on the feature importance is that, for time-domain features, RMS > LOG > AAC >> STD > MAV, and for frequency-domain features, PSR > MNP > MDF >> MNF > PKF. As the result, six features, RMS, LOG, AAC, PSR, MNP, and MDF were selected. After the feature datasets were calculated and selected by evaluating the feature importance, the obtained six feature values were input into the classifier. The average of accuracy with the six features slightly decreased by 0.7%, meanwhile, the computation time decreased by 30%.

### 4.2. Multi-Person Experiment

After the single person experiment, multi-person experiment was performed. In this experiment, the participants were required to perform each motion within three seconds, which corresponded to 600 pieces of EMG data. For each motion, the repeating cycle is set as 10. To verify the effectiveness of the bilinear model, data, with and without, the bilinear model process were input to the LSTM to test the accuracy.

#### 4.2.1. Classification without the Bilinear Model

With the feature calculation method mentioned in the previous section, the feature stacks from 19 participants were used for the training, and the features from one participant were used for the testing. This procedure repeats for 20 times as twenty-fold cross-validation with each time changing the testing participant until all the participants were tested once. 

The calculated features of 20 motions were directly input into the LSTM for classification. The classification result of 20 participants is shown in Table 4. The learning rate was set to 0.0001, and the iteration time was set to 500. 

The accuracy of 20 motions is shown in Table 5.

The accuracy of each participant is shown in Table 6.

#### 4.2.2. Classification with the Bilinear Model

From Table 5, almost all the motions cannot be correctly classified when the training data is not obtained from the test participant. What is more, from Table 6, the average classification accuracy of 20 motions among 20 participants drops sharply to 55.70%. As a result, it can be considered that the individual difference among different participants has strong influences on the EMG data so that the classification results are far from ideal. In order to solve the problem caused by the individual difference of the EMG data, as mentioned in Section 2.3, the bilinear model algorithm was applied. In this research, the user number ***U*** was 19 which was corresponding to the number of participants whose data was used for the training. The channel number ***C*** was set to 8 since there were 8 EMG sensors in the armband. The motion number ***M*** and the amount of data in each motion ***N*** were set at 20 and 10, respectively. The selection of the parameter ***I*** and ***J*** has a big influence on the final decomposition results since the values of the ***I*** and the ***J*** are the size of $z^{u}$, which contains the user factors, and the size of $x_{n}^{m}$, which contains the motion factors, respectively. 

**Figure 12:**
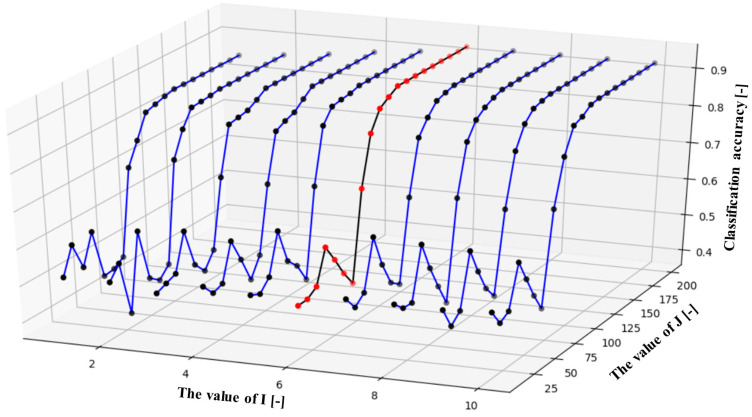
The influence of different ***I*** and ***J*** on the classification accuracy.

However, no theory or formula can describe how to decide the best ***I*** and ***J*** values. The choices of the ***I*** and the ***J*** were based on the prior experience, which means that the ***I*** and the ***J*** were adjusted according to the final classification accuracy. In this research, the range of the ***I*** was from 1 to 10, and the range of the ***J*** was from 10 to 200. Therefore, the results of different pairs of the ***I*** and the ***J*** were compared to select the most suitable one. The results of how the accuracy varies with different pairs of the ***I*** and the ***J*** are shown in Figure 12.

As shown in Figure 12, different pairs of the ***I*** and the ***J*** are compared to find the optimal choices. In Figure 12, the black curve with red points is the curve where the highest accuracy 90.5% occurs, of which the values of the ***I*** and the ***J*** are 6 and 120, respectively. Roughly, except for this line, the area of Figure 12 can be divided into three parts, which shows different situations of how the ***I*** and the ***J*** vary to influence the classification accuracy.

The first part is the lower part where the ***J*** is less than 80, no matter what value the ***I*** is, the classification accuracy was lower than 60%. It can be considered that when the ***J*** is lower than 80, the obtained motion matrix ***X*** does not contain enough motion factors for classification. This part is called the “lack of fitting” part.

The second part is the left top corner where the value of the **I** is less than 6 and the **J** is more than 120. In this case, accuracy was between 80% and 90%. It can be considered that the user factors cannot be completely separated from the EMG data; in other words, the obtained motion matrix ***X*** still had some user factors, which cause the accuracy was less than 90%. This part is called the “less suitable fitting” part.

The third part is the right top corner where the value of the ***I*** is more than 6 and the ***J*** is more than 120. In this case, the accuracy was almost the same which was from 87.5% to 90%. It can be concluded that almost all the user factors and the motion factors were well separated so that increasing the values of the ***I*** and the ***J*** had little influence on the accuracy, which is called as the “algorithm converged” part. 

**Figure 13:**
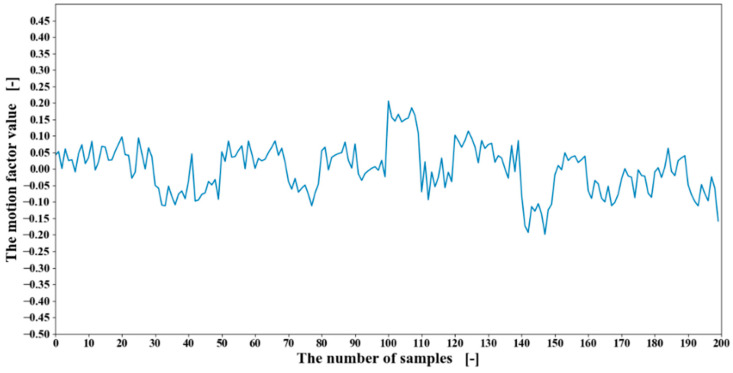
The extracted motion matrix factor values of participant one by the bilinear model.

Therefore, in this research, the value of the ***I*** and the ***J*** were set at 6 and 120, respectively. The extracted motion matrix factor values are shown in Figure 13. The motion matrix factor values were input into the LSTM for classification. The twenty-fold cross-validation with each time changing the testing participant until all the participants were tested. The learning rate was set to 0.0001, and the iteration time was set to 500. With the bilinear model applied, the accuracy of 20 participants, the results of the participant one, the results of 20 participants, and the accuracy of 20 motions are shown from Table 7, Table 8 and Table 9.

## 5. Discussion

**Figure 14:**
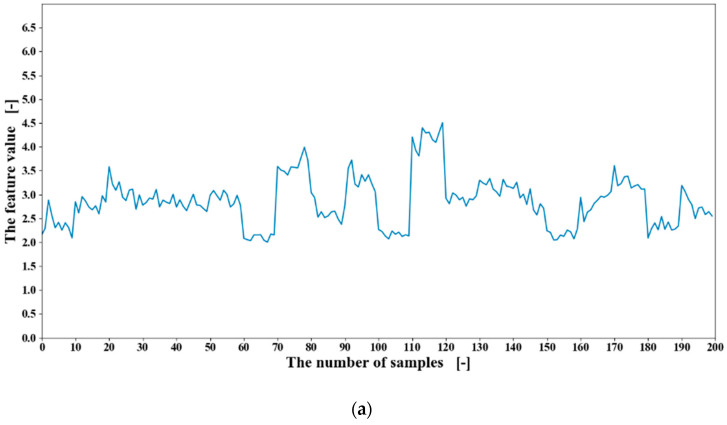

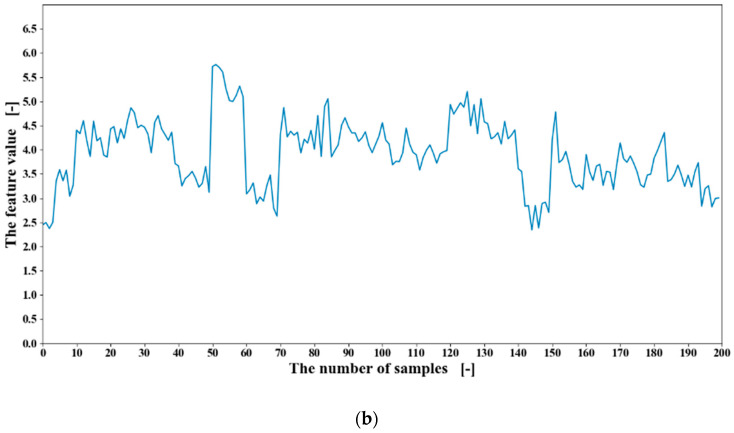
The RMS feature values of 20 motions from different participants:(**a**) participant four; (**b**) participant five.

**Figure 15:**
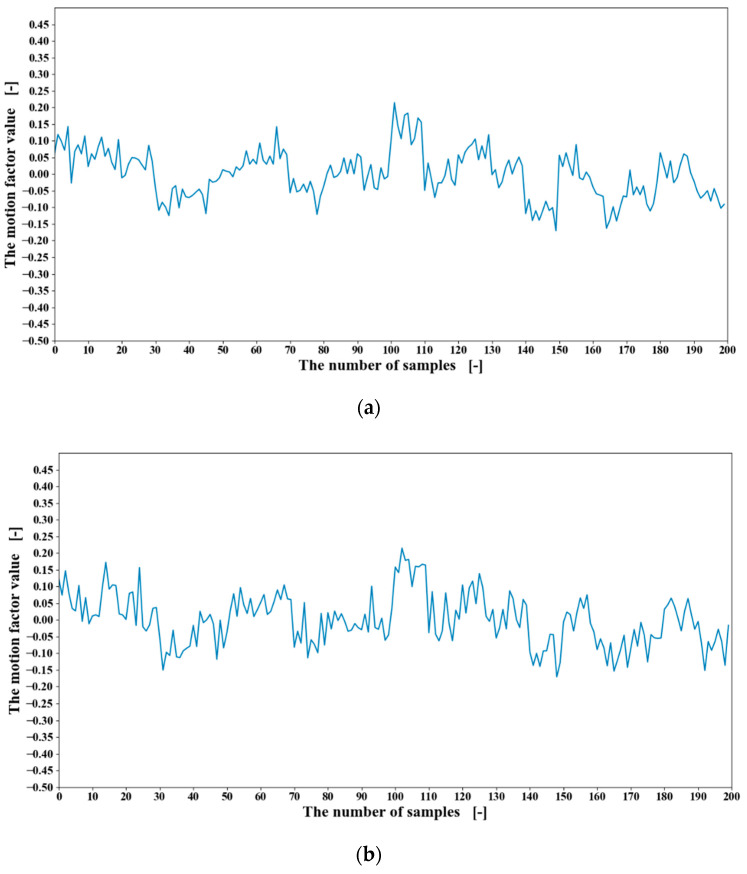
The motion matrix factor values of 20 motions from different participants; (**a**) participant four; (**b**) participant five.

To demonstrate the effectiveness of the bilinear model, the comparison of RMS feature values and motion factors from the other two participants were conducted as shown in Figure 14 and Figure 15. In Figure 14, graph (a) and graph (b) are of the same EMG data channel and the same feature, but from two participants. As shown in Figure 14, the RMS feature values of the two participants differ greatly. In contrast, after the introduction of the bilinear model, as shown in Figure 15, almost all the interferences of user factors are removed so that the values of the motion factors are much more similar. Although there are some differences between the motion factor values, it can be considered that the motion factors extracted from 19 participants are a little limited to represent the others’ motions.

In Table 6, participant three has the highest accuracy of 71.5%, and participant ten has the lowest accuracy of 46.5%. The accuracy difference between the two participants is 25%, which can indicate the existence of the individual difference. In Table 9, participant four and twelve have the highest accuracy of 100%, and participant sixteen has the lowest accuracy of 94.5%. The accuracy difference among the participants has decreased to 5.5%. Moreover, the average accuracy has increased to 99.7%. Compared with the results of Table 6 and Table 9, by applying the bilinear model algorithm, the influence of the individual difference has been largely decreased, which shows that the bilinear model is very effective in the decomposition of user factors and motion factors.

In Table 5, motion six has the highest accuracy of 63.0%, which means that the motions are barely recognized. In Table 8, motion eight and nine have the highest accuracy of 99.5%, and motion ten has the lowest accuracy of 94.0%. The accuracy difference is 5.5%. It can be concluded that almost all the 20 motions are well classified. Misjudgments can be considered that they are mostly caused by the similarities among the 20 motions. For motion ten, the reason it has more misjudgments than the other motions is that it includes more sequential gestures than the others. Another reason can be that the user factors cannot be completely removed and the motion factors are just representative among the 19 participants, which means there are still some differences between the training motion matrix and the testing motion matrix. If more motion data can be obtained from different people, the influence of the individual difference will be no longer a significant problem that has influence on the classification accuracy.

The Myo band has a sampling rate of 200 Hz, which in some cases, is not enough to obtain all the information of the EMG signal. However, in this research, with an accuracy of 99.7%, it is reasonable to address that this limitation of sampling rate has little impact on this system.

Table 10 shows the performance and characteristics of our proposed system and other studies using the Myo band as a sensor device [61]. Even though the performance metrics of these studies cannot be compared directly due to different experiment settings, they are helpful for qualitative comparisons. Among them, the studies of [43,62] had the high accuracy more than 99%; however, the number of recognized gestures was less than 10, and [62] needed about 1 s to perform the recognition. On the other hand, although the study of [42] needed only 3 ms, the accuracy is 85.1%. Compared with other studies, our proposed system can recognize 20 sign language motions with 97.7% accuracy among 20 participants, and also can perform a real-time recognition with a delay time of less than 50 ms on a PC platform with Intel Core-i7 3.2 GHz and no GPU. Therefore, our system has the potential to be widely applied, and may be implemented into smart phones to realize a real-time daily conversation system by hand gestures for hearing-impaired people.

## 6. Conclusions

This paper presented a user-independent sign language motion recognition system based on the electromyography signal for both helping hearing-impaired people communicate with others more easily in their daily life and training normal people to understand the sign language motions. This proposed motion recognition system could recognize 20 meaningful and widely used ASL motions with high accuracy.

In this paper, the characteristic of the EMG signal was analyzed and utilized for motion recognition. The EMG signal itself is a strong indicator representing the muscle movements; however, it has obvious individual differences among multiple people. Therefore, in this research, the bilinear model algorithm was applied to obtain motion factors for classification. With the introduction of the bilinear model, the interferences of user factors were largely decreased and the motion factors were extracted for classification. Finally, the LSTM was used as the classifier of motions. Moreover, the permutation importance of the features was performed to select the most important features to reduce computation time-consuming. As a result, the LSTM with the bilinear model could realize real-time hand gesture recognition with very high accuracy among 20 participants.

## Figures and Tables

**Table 1:** The 20 hand motions.

Motion No.	Motion Name	Motion No.	Motion Name
M1	How are you?	M11	Where is the store?
M2	Nice to meet you.	M12	How can I get food?
M3	See you later.	M13	How much does it cost?
M4	That’s what I mean.	M14	Yes, thank you.
M5	I don’t understand.	M15	I am sorry.
M6	What is your name?	M16	Where is the hospital?
M7	Where are you from?	M17	I don’t feel good.
M8	What happens?	M18	Please help me.
M9	What is wrong?	M19	Please write it.
M10	Please call 911.	M20	I love you.

**Table 2:** The classification result of the LSTM on the single participant.

	Predicted Label																				
**Actual Label**		M1	M2	M3	M4	M5	M6	M7	M8	M9	M10	M11	M12	M13	M14	M15	M16	M17	M18	M19	M20
	M1	**47**	0	2	0	0	0	0	0	0	0	0	0	0	0	1	0	0	0	0	0
	M2	0	**49**	0	0	0	0	0	1	0	0	0	0	0	0	0	0	0	0	0	0
	M3	1	0	**47**	0	0	0	1	0	0	0	0	0	0	0	1	0	0	0	0	0
	M4	0	0	0	**48**	0	0	0	2	0	0	0	0	0	0	0	0	0	0	0	0
	M5	0	0	1	0	**49**	0	0	0	0	0	0	0	0	0	0	0	0	0	0	0
	M6	0	0	0	0	0	**49**	0	0	0	1	0	0	0	0	0	0	0	0	0	0
	M7	0	0	0	0	0	0	**49**	0	0	0	1	0	0	0	0	0	0	0	0	0
	M8	0	0	0	1	0	0	0	**47**	0	0	0	0	1	0	0	1	0	0	0	0
	M9	0	0	0	0	0	0	0	0	**50**	0	0	0	0	0	0	0	0	0	0	0
	M10	0	0	0	0	0	0	0	0	0	**50**	0	0	0	0	0	0	0	0	0	0
	M11	0	0	0	0	0	0	1	0	0	0	**49**	0	0	0	0	0	0	0	0	0
	M12	0	0	0	0	0	0	0	0	0	0	0	**49**	0	0	0	0	0	0	1	0
	M13	0	0	0	0	0	1	0	0	0	0	0	0	**49**	0	0	0	0	0	0	0
	M14	0	0	0	0	0	0	0	0	0	0	0	2	0	**48**	0	0	0	0	0	0
	M15	0	0	0	0	1	0	0	0	0	0	0	0	0	0	**49**	0	0	0	0	0
	M16	0	0	0	0	0	0	0	0	0	0	0	0	0	0	0	**50**	0	0	0	0
	M17	0	0	0	0	0	0	0	0	0	0	0	1	0	0	0	0	**49**	0	0	0
	M18	0	0	0	0	0	0	0	0	0	0	0	0	0	0	0	0	0	**50**	0	0
	M19	0	0	0	0	0	0	0	0	0	0	0	0	0	0	0	0	0	0	**50**	0
	M20	0	0	0	0	0	0	0	0	0	1	0	0	0	0	1	0	0	0	0	**48**

**Table 3:** The importance of selected ten features.

	Time-Domain Features					Frequency-Domain Features				
	MAV	STD	RMS	LOG	AAC	MNF	MDF	MNP	PSR	PKF
Accuracy	98.54%	97.38%	77.34%	87.68%	89.74%	97.94%	88.48%	86.52%	81.72%	96.30%
Importance	1.32%	2.48%	22.52%	12.18%	10.12%	2.92%	11.42%	14.34%	19.14%	3.56%

**Table 4:** The total classification results of 20 participants.

	Predicted Label																				
**Actual Label**		M1	M2	M3	M4	M5	M6	M7	M8	M9	M10	M11	M12	M13	M14	M15	M16	M17	M18	M19	M20
	M1	**101**	2	5	1	3	7	7	4	10	3	5	4	8	1	11	6	7	6	4	5
	M2	6	**107**	3	3	6	7	2	4	6	6	3	5	3	6	6	4	4	8	5	6
	M3	6	11	**117**	3	7	4	4	5	3	8	4	3	2	4	1	4	4	3	4	3
	M4	6	4	7	**109**	4	4	1	7	3	4	3	5	5	2	4	1	9	7	5	10
	M5	7	5	3	2	**116**	2	5	8	4	4	1	2	4	3	3	1	5	8	9	8
	M6	4	2	9	1	4	**126**	8	2	3	3	5	3	5	3	4	3	2	6	3	4
	M7	6	5	2	4	5	7	**113**	7	6	1	5	4	4	1	6	5	7	5	4	3
	M8	5	1	7	4	3	8	1	**120**	4	1	3	3	2	6	1	6	5	15	2	3
	M9	4	5	4	3	3	0	9	2	**112**	4	3	4	2	2	3	6	8	8	12	6
	M10	5	5	7	8	7	4	7	4	4	**104**	4	4	3	3	3	10	2	8	4	4
	M11	5	5	2	5	7	6	11	3	4	7	**103**	1	2	5	2	3	5	8	7	9
	M12	6	4	5	4	4	8	3	4	3	6	4	**107**	3	5	7	5	5	6	6	5
	M13	3	3	5	4	5	5	7	7	9	5	3	4	**98**	4	1	9	6	6	7	9
	M14	5	4	5	6	3	2	8	6	2	3	4	3	3	**122**	1	2	3	6	6	6
	M15	5	2	5	3	7	2	11	2	6	5	3	1	5	6	**115**	5	3	7	3	4
	M16	4	3	8	4	8	3	4	1	4	2	5	3	4	9	5	**116**	1	2	7	7
	M17	5	6	4	10	3	5	6	3	4	5	4	2	6	2	3	8	**108**	9	6	1
	M18	4	4	3	4	3	5	4	10	2	3	4	3	6	4	8	3	6	**117**	3	4
	M19	4	2	4	5	4	5	8	4	10	2	2	4	7	6	3	2	4	4	**115**	5
	M20	9	4	3	5	8	1	7	2	7	7	2	4	8	3	5	2	6	7	6	**104**

**Table 5:** The classification accuracy of 20 hand motions on 20 participants.

Motion Name	Accuracy	Motion Name	Accuracy
M1: How are you?	50.5%	M11: Where is the store?	51.5%
M2: Nice to meet you.	53.5%	M12: How can I get food?	53.5%
M3: See you later.	58.5%	M13: How much does it cost?	49.0%
M4: That’s what I mean.	54.5%	M14: Yes, thank you.	61.0%
M5: I don’t understand.	58.0%	M15: I am sorry.	57.5%
M6: What is your name?	63.0%	M16: Where is the hospital?	58.0%
M7: Where are you from?	56.5%	M17: I don’t feel good.	54.4%
M8: What happens?	60.0%	M18: Please help me.	58.5%
M9: What is wrong?	56.0%	M19: Please write it.	57.5%
M10: Please call 911.	52.0%	M20: I love you.	52.0%

**Table 6:** The classification accuracy of 20 participants.

Name	Accuracy	Name	Accuracy
Participant 1	60.5%	Participant 11	49.5%
Participant 2	50.5%	Participant 12	62.0%
Participant 3	71.5%	Participant 13	48.0%
Participant 4	61.0%	Participant 14	61.0%
Participant 5	49.0%	Participant 15	61.5%
Participant 6	64.0%	Participant 16	59.5%
Participant 7	54.0%	Participant 17	50.0%
Participant 8	49.5%	Participant 18	54.5%
Participant 9	57.5%	Participant 19	51.5%
Participant 10	46.5%	Participant 20	48.0%

**Table 7:** The classification accuracy of 20 participants with the bilinear model.

	Predicted Label																				
**Actual Label**		M1	M2	M3	M4	M5	M6	M7	M8	M9	M10	M11	M12	M13	M14	M15	M16	M17	M18	M19	M20
	M1	**196**	0	0	1	0	0	2	0	0	0	0	0	0	0	0	0	0	1	0	0
	M2	0	**197**	0	0	0	1	1	0	0	0	0	0	0	0	0	0	1	0	0	0
	M3	0	1	**198**	0	1	0	0	0	0	0	0	0	0	0	0	0	0	0	0	0
	M4	0	0	1	**194**	1	0	0	1	0	0	0	1	0	0	0	0	2	0	0	0
	M5	0	0	0	0	**194**	0	0	0	0	0	0	2	0	0	0	0	0	2	0	2
	M6	0	1	0	0	0	**197**	0	0	0	1	0	0	0	0	0	1	0	0	0	0
	M7	0	0	0	0	0	1	**195**	0	0	1	0	1	0	0	0	1	0	0	0	1
	M8	0	0	0	0	0	1	0	**199**	0	0	0	0	0	0	0	0	0	0	0	0
	M9	0	0	0	0	0	0	0	0	**199**	0	0	0	0	0	0	0	1	0	0	0
	M10	0	1	1	0	1	3	1	0	1	**188**	0	0	1	1	0	0	0	0	2	0
	M11	0	0	0	0	0	0	0	0	0	0	**196**	0	0	1	0	1	0	0	1	1
	M12	0	2	0	0	0	0	0	1	0	0	0	**196**	0	0	0	0	0	1	0	0
	M13	0	0	1	0	0	0	0	0	1	0	0	0	**198**	0	0	0	0	0	0	0
	M14	0	0	0	0	0	0	0	0	1	0	0	0	1	**198**	0	0	0	0	0	0
	M15	0	0	1	0	1	0	0	0	0	0	0	0	1	0	**193**	0	0	2	0	2
	M16	0	1	0	0	0	0	0	0	0	0	0	0	0	0	0	**198**	0	0	0	1
	M17	0	1	0	1	0	1	0	0	0	0	0	0	0	0	0	0	**194**	1	1	1
	M18	0	0	1	0	1	1	0	0	0	0	0	0	0	0	2	0	1	**194**	0	0
	M19	0	0	0	0	0	1	0	0	1	0	0	0	1	0	0	0	0	1	**196**	0
	M20	0	0	0	0	1	1	0	0	0	0	0	1	0	1	4	1	0	0	1	**190**

**Table 8:** The classification accuracy of 20 motions with the bilinear model.

Motion Name	Accuracy	Motion Name	Accuracy
M1: How are you?	98.0%	M11: Where is the store?	98.0%
M2: Nice to meet you.	98.5%	M12: How can I get food?	98.0%
M3: See you later.	99.0%	M13: How much does it cost?	99.0%
M4: That’s what I mean.	97.0%	M14: Yes, thank you.	99.0%
M5: I don’t understand.	97.0%	M15: I am sorry.	96.5%
M6: What is your name?	98.5%	M16: Where is the hospital?	99.0%
M7: Where are you from?	97.5%	M17: I don’t feel good.	97.0%
M8: What happens?	99.5%	M18: Please help me.	97.0%
M9: What is wrong?	99.5%	M19: Please write it.	98.0%
M10: Please call 911.	94.0%	M20: I love you.	95.0%

**Table 9:** The classification accuracy of 20 participants with the bilinear model.

Name	Accuracy	Name	Accuracy
Participant 1	98.5%	Participant 11	95.0%
Participant 2	98.0%	Participant 12	100.0%
Participant 3	99.0%	Participant 13	95.0%
Participant 4	100.0%	Participant 14	98.0%
Participant 5	96.5%	Participant 15	98.5%
Participant 6	98.5%	Participant 16	94.5%
Participant 7	99.0%	Participant 17	95.5%
Participant 8	97.5%	Participant 18	98.0%
Participant 9	98.5%	Participant 19	99.0%
Participant 10	98.0%	Participant 20	98.0%

**Table 10:** Comparison with other studies using a Myo band.

Study	RTP (ms)	Gestures	Duration (s)	Participants	Repetition	Classifier	Accuracy (%)
Savur [22]	NI	27	2	10	20	SVM	60.9
Hu [34]	NI	52	5	27	10	LCNN	87.0
Kerber [41]	500	5	NI	14	NI	SVM	95.0
Chung [42]	3	5	5	120	50	ANN	85.1
Raurale [43]	4.5/8.8	9	5	10	20	RBF	99.0
Zhang [61]	200	21	2	13	30	GRU	89.6
Nasri [62]	940	6	10	35	195	GRU	99.8
Ali [63]	NI	18	5	40	6	LSTM	89.5
He [64]	400	52	5	27	10	LSTM	75.5
Ours	50	20	3	20	10	BL + LSTM	97.7

Note: RTP represents real-time performance. NI means the corresponding term is not indicated in the paper clearly. LCNN is the combination of LSTM and CNN. ANN is an artificial neural network. RBF is a radial basis function neural network. GRU means gated recurrent units. BL means a bilinear model.
